# Development and internal validation of the elderly COPD diagnostic score (ECDS): a multidimensional diagnostic tool for moderate-to-severe chronic obstructive pulmonary disease

**DOI:** 10.3389/fmed.2026.1770654

**Published:** 2026-03-27

**Authors:** Junmin Li, Quanzhu Fu, Hui Xiao

**Affiliations:** 1 Department of Pulmonary and Critical Care Medicine, Renmin Hospital, Hubei University of Medicine, Shiyan, Hubei, China; 2 Department of Critical Care Medicine, Taihe Hospital, Hubei University of Medicine, Shiyan, Hubei, China

**Keywords:** chronic obstructive pulmonary disease, comorbidity, diagnosis, elderly, multidimensional score, spirometry, validation

## Abstract

**Background:**

Accurate diagnosis of chronic obstructive pulmonary disease (COPD) among elderly individuals (≥65 years) presents considerable clinical difficulties. Age-associated physiological alterations, frequent multimorbidity, and atypical symptom manifestations complicate the diagnostic process. The conventional fixed FEV₁/FVC threshold of <0.70 may not be optimally suited for this population. This study aimed to construct and validate a novel multidimensional diagnostic tool that integrates both clinical and functional parameters.

**Methods:**

We conducted a single-center retrospective diagnostic accuracy study involving 976 symptomatic elderly patients (mean age 73.0 ± 6.7 years) who underwent comprehensive post-bronchodilator spirometry between February 2023 and March 2025. A blinded multidisciplinary expert panel established final diagnoses (moderate/severe COPD versus non-COPD) based on GOLD 2023 criteria and comprehensive clinical assessment. Using a temporal split approach, patients were allocated to either a Derivation Cohort (*n* = 650, February–December 2023) or an independent Internal Validation Cohort (*n* = 326, January 2024–March 2025). Within the Derivation Cohort, we developed the Elderly COPD Diagnostic Score (ECDS) through multivariable logistic regression and established age-specific FEV₁/FVC diagnostic thresholds. The finalized ECDS formula and threshold values were subsequently applied to the Validation Cohort without modification. Diagnostic performance was evaluated using ROC-AUC analysis, sensitivity and specificity calculations, and decision curve analysis (DCA).

**Results:**

The ECDS incorporates five weighted components: (100 - FEV₁/FVC), age >65 years, Charlson Comorbidity Index, mMRC dyspnea scale score, and COPD Assessment Test score. In the Derivation Cohort, the ECDS demonstrated excellent discriminative ability with an AUC of 0.972 (95% CI: 0.962–0.982), significantly outperforming FEV₁/FVC alone (AUC 0.942, *p* < 0.05). At the optimal cutoff of ≥2.8, sensitivity reached 96.4% with specificity of 93.2%. In the independent Validation Cohort, the ECDS maintained robust performance with an AUC of 0.968, sensitivity of 95.1%, and specificity of 90.8%. DCA confirmed superior net clinical benefit across relevant threshold probabilities compared to alternative diagnostic strategies. Age-stratified FEV₁/FVC cutoffs (e.g., <64.5% for age ≥80) proved more accurate than the fixed <70% threshold. Notably, diagnostic accuracy of standard FEV₁/FVC measurement significantly diminished among patients with high comorbidity burden (CCI ≥ 5).

**Conclusion:**

The Elderly COPD Diagnostic Score (ECDS), which synthesizes spirometric data, clinical parameters, and comorbidity information, demonstrated excellent and validated diagnostic accuracy for moderate-to-severe COPD in elderly patients, surpassing the performance of conventional FEV₁/FVC criteria. Implementation of age-specific spirometric thresholds further refines diagnostic precision. The ECDS represents a practical, superior tool for diagnosing COPD in the complex geriatric patient population. While the ECDS offers a superior diagnostic tool for moderate-to-severe COPD in older adults, its performance in mild disease requires further validation.

## 1 Introduction

Chronic obstructive pulmonary disease (COPD) ranks among the foremost contributors to illness and death worldwide. Among individuals aged 65 years and older, the occurrence, intensity, and overall impact of this condition surge markedly, evolving into a pressing public health concern (1, 2). Precise identification forms the bedrock of suitable treatment strategies, yet securing an accurate diagnosis in older adults continues to pose a considerable clinical hurdle (3).

The prevailing diagnostic benchmark, as outlined by the Global Initiative for Chronic Obstructive Lung Disease (GOLD), depends on demonstrating persistent airflow limitation through a post-bronchodilator forced expiratory volume in one second to forced vital capacity (FEV₁/FVC) ratio below 0.70 (4). However, the practice of utilizing this rigid, unchanging cutoff point for all elderly patients is now facing growing doubt and critical examination (5). The natural aging process brings about multifaceted changes in respiratory physiology, including loss of lung elastic recoil and greater rigidity of the chest wall. These changes can precipitate a decline in FEV₁/FVC that falls within the expected range for healthy aging (6, 7). As a result, adhering strictly to the fixed ratio might incorrectly label some healthy older persons with a COPD diagnosis (8, 9). On the other hand, the symptom profile of COPD in the elderly is often non-specific, easily obscured by the presence of multiple other chronic conditions (multimorbidity), complex medication regimens, and general age-related health syndromes. This scenario significantly increases the likelihood of missing the diagnosis (10, 11). Furthermore, common co-existing ailments such as chronic heart failure, interstitial lung diseases, or bronchiectasis add layers of complexity to distinguishing COPD from other causes of respiratory symptoms (12). Even the cornerstone test, spirometry, presents practical difficulties for certain older individuals. Cognitive, sensory, or physical impairments can hinder their ability to perform the test optimally, which may compromise the quality and reliability of the results (13, 14).

A growing body of research points to the shortcomings of relying on a single diagnostic parameter. Alternative methods, like employing statistically defined lower limits of normal (LLN) or age-specific percentile cutoffs for FEV₁/FVC, have been proposed. Nonetheless, these approaches lack universal agreement and are not commonly integrated into standard clinical workflows (15, 16). Compounding this issue is the high prevalence of accompanying health problems in the elderly. Conditions such as cardiovascular disease and metabolic syndrome are frequent in older COPD patients and can themselves cause symptoms like breathlessness and reduced exercise capacity, thereby muddying the diagnostic waters (17, 18). This context highlights a potential gap: the need for a diagnostic framework that adopts a broader perspective. Such an approach would merge spirometric data with other easily obtainable clinical metrics, including validated symptom questionnaires, indices of comorbidity burden, and assessments of functional status (19, 20).

Although several models and scores are available for predicting the future course of COPD or estimating risk, very few have been purpose-built and thoroughly tested for the initial diagnosis of moderate-to-severe disease specifically in the geriatric population (21, 22). Consequently, devising a simple, unified diagnostic instrument that synthesizes essential clinical and functional information could potentially enhance diagnostic precision and promote earlier treatment initiation in this susceptible group.

Driven by these considerations, the present study set out to accomplish three main goals: firstly, to create and perform an internal validation of a new multidimensional diagnostic score—the Elderly COPD Diagnostic Score (ECDS)—for detecting moderate-to-severe COPD in symptomatic older patients; secondly, to determine and validate FEV₁/FVC diagnostic thresholds tailored to different age strata within this population; and thirdly, to investigate how the weight of comorbid conditions affects the diagnostic accuracy of conventional spirometry.

## 2 Materials and methods

### 2.1 Study design and setting

This research followed a diagnostic accuracy framework with retrospective data collection at a single institution, incorporating internal validation through temporal cohort separation. The investigation was carried out within the Respiratory Medicine Department of our hospital. Data collection spanned a consecutive 26-month interval from February 1, 2023, through March 31, 2025. The central aim was to create, refine, and subsequently validate a new composite scoring mechanism for reliably detecting moderate-to-severe COPD in symptomatic patients aged 65 years or older. Study procedures conformed to the STARD (Standards for Reporting Diagnostic Accuracy) guidelines. Ethical approval was obtained from Taihe Hospital Review Board (TH-rp-022-09-35). Given the retrospective design and use of anonymized patient information, the ethics committee granted a waiver for obtaining individual informed consent.

### 2.2 Participant eligibility and screening process

Potential participants were identified through a systematic search of the hospital’s unified electronic medical record system. Our screening targeted all consecutive patients aged 65 years or above who presented to the respiratory outpatient clinic or were hospitalized under respiratory services during the study period, and who had undergone comprehensive pulmonary function testing as part of diagnostic evaluation. The initial electronic query identified 1,586 records meeting these preliminary criteria.

#### 2.2.1 Inclusion criteria

Patients were included if they met all the following conditions: (1) aged 65 years or older at pulmonary function testing; (2) exhibited chronic respiratory symptoms (dyspnea, persistent cough, sputum production, or wheezing) for at least 3 months; (3) possessed complete standardized clinical data including smoking history (pack-years), biomass exposure duration, occupational exposure status, Charlson Comorbidity Index score, mMRC dyspnea scale and CAT symptom scores, exacerbation history in the preceding 12 months, 6-min walk distance test result, chest CT scan report, and at least 6 months of clinical follow-up; and (4) successfully completed post-bronchodilator spirometry meeting 2019 ATS/ERS quality standards.

#### 2.2.2 Exclusion criteria

Patients were excluded if they met any of the following conditions: (1) Experienced an acute exacerbation of their respiratory condition requiring systemic corticosteroids and/or antibiotics within 4 weeks prior to PFT. (2) Had a history of major lung resection surgery (lobectomy or pneumonectomy), active pulmonary tuberculosis, or a current diagnosis of lung cancer. (3) Presented with neuromuscular diseases (e.g., amyotrophic lateral sclerosis, myasthenia gravis) or significant thoracic skeletal deformities (e.g., severe kyphoscoliosis) known to severely compromise the ability to perform reliable spirometry. (4) Had missing or technically inadequate data for any of the key variables listed in the inclusion criteria. (5) Received a final, consensus diagnosis of a specific respiratory condition other than COPD that fully explained their symptoms, such as bronchial asthma, interstitial lung disease, bronchiectasis, pulmonary hypertension, or congestive heart failure.

This study focused exclusively on patients with moderate-to-severe COPD (GOLD grades 2 and 3) for two primary reasons. First, the diagnostic challenges in elderly patients are most pronounced when airflow limitation is sufficiently advanced to cause significant symptoms and functional impairment, yet remains confounded by age-related physiological decline and multimorbidity (8, 10). Second, the clinical reference standard—based on expert panel adjudication using longitudinal follow-up data—is most reliable when the distinction between COPD and non-COPD is clear; mild obstruction (GOLD grade 1) often presents with non-specific symptoms and may overlap with normal age-related lung function changes, increasing the risk of diagnostic misclassification (5, 15). By restricting the study population to moderate-to-severe cases, we aimed to ensure a clean and robust reference standard, thereby enhancing the internal validity of the ECDS.

### 2.3 Reference standard and diagnostic adjudication

To establish the reference standard, an independent multidisciplinary committee (two senior pulmonologists and one cardiothoracic radiologist) reviewed all clinical data, including medical history, physical examination findings, high-resolution chest CT images, and at least 6 months of follow-up information. Crucially, the committee members were initially blinded to all spirometric values, including FEV₁, FVC, and the FEV₁/FVC ratio. Based solely on the non-spirometric data, they rendered a preliminary diagnosis, classifying each patient as either “COPD” or “non-COPD.”

Only after this preliminary classification was recorded were the post-bronchodilator spirometry results disclosed. These results were then used to grade the severity of COPD according to the GOLD 2023 criteria (moderate: FEV₁% predicted 50–79%; severe: FEV₁% predicted <50%) among those preliminarily diagnosed with COPD. This two-step adjudication process was specifically designed to prevent incorporation bias, ensuring that the reference standard (COPD vs. non-COPD) remained independent of the predictor variables used in the ECDS.

The detailed two-step adjudication process is illustrated in Supplementary Figure S1.

### 2.4 Data abstraction and variable definitions

A trained research team under principal investigator supervision extracted data using a pre-piloted electronic form in REDCap to ensure standardization. Collected variables included: demographic characteristics (age, sex, BMI); risk factors (smoking pack-years, biomass exposure duration, occupational exposure history); comorbidities (Charlson Comorbidity Index derived from ICD-10 codes, plus specific cardiometabolic conditions documented as binary variables); clinical status indicators (symptom duration, mMRC dyspnea scale, CAT score, exacerbation frequency in past year, 6-min walk distance); and pulmonary function parameters (FEV₁, FVC, FEV₁/FVC ratio, FEV₁% predicted, FVC% predicted) obtained from post-bronchodilator maneuvers meeting quality standards.

### 2.5 Pulmonary function testing protocol

All spirometry testing occurred in the hospital’s accredited Pulmonary Function Laboratory using calibrated flow-sensing spirometers (MasterScreen PFT System, CareFusion/Jaeger, Germany) with daily quality control procedures. Certified technicians administered tests following 2019 ATS/ERS technical standards, with patients seated and wearing nose clips. The protocol included pre-bronchodilator maneuvers (minimum three attempts to obtain three acceptable and two reproducible curves), administration of 400 μg salbutamol via spacer device, and post-bronchodilator testing after a precise 20-min interval, again requiring three acceptable and two reproducible maneuvers.

### 2.6 Derivation and validation cohort design

To minimize overfitting and ensure rigorous evaluation, we implemented a temporal cohort split strategy. The Derivation Cohort included all eligible patients from the initial 11-month period (February–December 2023, *n* = 650) and was used exclusively for model development and initial cutoff determination. The independent Internal Validation Cohort comprised patients from the subsequent 15-month period (January 2024–March 2025, *n* = 326) and was reserved solely for unbiased validation of models and thresholds derived from the derivation phase. Baseline characteristics between cohorts were compared to confirm comparability (Supplementary Table 1).

### 2.7 Derivation of novel diagnostic parameters (performed on derivation cohort)

To move beyond single-parameter diagnostics, we developed two composite metrics using data from the Derivation Cohort only.

1) FEV₁/FVC Ratio Index Development:

To address the limitations of single-parameter diagnostics, a novel composite metric—the FEV₁/FVC Ratio Index—was developed within the Derivation Cohort. This index was specifically designed to provide an intuitive integration of both obstruction severity and degree through a mathematically defined calculation: Index = [(100 – FEV₁/FVC) × FEV₁% predicted] / 100. By simultaneously incorporating both airflow limitation (measured by the deviation from normal FEV₁/FVC) and the overall functional impairment (represented by FEV₁% predicted), the index generates higher numerical values that directly correlate with more severe obstructive lung disease presentation, thereby offering a more comprehensive quantitative assessment of COPD severity than either parameter alone.

2) Elderly COPD Diagnostic Score Development:

The development of the Elderly COPD Diagnostic Score (ECDS) followed a systematic three-phase approach. Initial predictor selection considered clinically relevant variables including age, Charlson Comorbidity Index (CCI), mMRC dyspnea scale, COPD Assessment Test (CAT) score, 6-min walk distance (6MWD), exposure indices, and the core parameter of airflow obstruction (100 – FEV₁/FVC). Subsequently, multivariable logistic regression modeling was performed with moderate-to-severe COPD diagnosis versus non-COPD controls as the binary outcome variable. Variable selection employed backward elimination methodology with a P-to-remove threshold greater than 0.10, while maintaining clinical relevance considerations. The final score creation involved standardizing significant regression coefficients and applying a transformation process: each coefficient was divided by the smallest statistically significant coefficient and multiplied by a scaling factor to generate integer-friendly weights, resulting in the clinically practical formula: ECDS = 0.5 × (100 – FEV₁/FVC) + 0.2 × (Age – 65) + 0.15 × CCI + 0.1 × mMRC + 0.05 × (10 – CAT/2.5). Notably, age was centered at 65 years, and the CAT score transformation ensured all components positively contributed to the COPD-predictive score value.

### 2.8 Statistical analysis

Analyses were performed using SPSS Statistics 26.0 (IBM Corp.) and R 4.3.0 (R Foundation). A two-sided *p*-value < 0.05 defined statistical significance.

#### 2.8.1 Descriptive and comparative statistics

Continuous variables are presented as mean ± standard deviation (SD) if normally distributed (assessed by Shapiro–Wilk test), or median (interquartile range) if not. Categorical variables are presented as counts and percentages (*n*, %). Comparisons across the three diagnostic groups (Severe COPD, Moderate COPD, Non-COPD) were made using one-way ANOVA (with post-hoc Tukey test) or the Kruskal-Wallis H test for continuous variables, and the Chi-square test or Fisher’s exact test for categorical variables. The balance between the Derivation and Validation cohorts was assessed using independent *t*-tests or Mann–Whitney *U* tests, and chi-square tests.

Diagnostic Accuracy Analysis (Conducted Separately by Cohort).

Diagnostic accuracy assessment was performed independently for each cohort. Receiver operating characteristic (ROC) curves were generated for five key parameters against the established reference standard: the conventional FEV₁/FVC ratio, FEV₁% predicted, FVC% predicted, the novel FEV₁/FVC Ratio Index, and the newly developed ECDS. For each diagnostic parameter, we computed comprehensive performance metrics including the area under the curve (AUC) with its 95% confidence interval, sensitivity, specificity, positive and negative predictive values, and positive and negative likelihood ratios. Optimal diagnostic thresholds were determined by identifying the cutoff value that maximized Youden’s Index (J = Sensitivity + Specificity − 1) within the Derivation Cohort. Statistical comparison of AUC values among different parameters within each cohort was performed using the DeLong test.

#### 2.8.2 Model development and internal validation

The logistic regression model underlying the ECDS underwent comprehensive evaluation for both discrimination (quantified by AUC) and calibration. Calibration assessment employed visual inspection of calibration plots alongside formal statistical testing using the Hosmer-Lemeshow goodness-of-fit test. For internal validation, a strict locking procedure was implemented: the finalized ECDS formula and all optimal diagnostic cutoffs (for FEV₁/FVC, the Ratio Index, and the ECDS itself) derived exclusively from the Derivation Cohort were fixed. These locked parameters were then directly applied, without any adjustment or recalibration, to the independent Validation Cohort. Performance metrics were subsequently recalculated in this validation dataset to objectively assess generalizability and quantify potential optimism in the model’s performance estimates.

To further assess the potential for overfitting and to obtain optimism-corrected performance estimates, we performed internal validation using bootstrapping with 1,000 resamples within the Derivation Cohort. For each bootstrap sample, the entire model development process (including variable selection and coefficient estimation) was repeated, and the apparent performance was compared with the performance in the original sample. The average optimism was calculated and subtracted from the apparent AUC to derive the optimism-corrected AUC.

In addition, as a sensitivity analysis, we refitted the multivariable logistic regression model using penalized maximum likelihood estimation (LASSO regression) with 10-fold cross-validation to select the regularization parameter (*λ*). This approach shrinks regression coefficients toward zero, reducing overfitting and enhancing generalizability. The performance of the LASSO-derived model was evaluated in both the Derivation and Validation Cohorts.

#### 2.8.3 Clinical utility and decision analysis

Decision Curve Analysis (DCA): Performed in both cohorts to evaluate the net clinical benefit of diagnostic strategies (e.g., “diagnose all,” “diagnose none,” using FEV₁/FVC cutoff, using ECDS cutoff) across a range of clinically relevant threshold probabilities (10 to 50%).

#### 2.8.4 Exploratory and sensitivity analyses

Correlation Analysis: Pairwise Pearson correlation coefficients among key continuous variables (e.g., FEV₁/FVC, mMRC, CAT, 6MWD, CCI, Age) were calculated for the total population and visualized as a correlation matrix heatmap.

Network Analysis: Using the qgraph package in R, a partial correlation network was constructed to visualize the conditional dependence relationships among variables. Betweenness centrality and closeness centrality metrics were computed to identify the most influential “hub” variables in the clinical network of COPD.

Stratified Analysis (Age): A stratified analysis based on age was conducted to evaluate how diagnostic thresholds for FEV₁/FVC might systematically vary across the older adult population. Within the Derivation Cohort, participants were categorized into four age groups: 65 to 69 years, 70 to 74 years, 75 to 79 years, and those aged 80 years or older. For each of these age-specific strata, an optimal FEV₁/FVC cutoff point that maximized diagnostic accuracy for that particular age bracket was calculated. The performance of these newly derived, age-decade-specific cutoffs was then evaluated independently, first within the original Derivation Cohort and subsequently applied to the separate Validation Cohort to assess their generalizability and robustness.

Subgroup Analysis (Comorbidity): To investigate whether the presence of other chronic conditions (comorbidities) influences the accuracy of standard diagnostic methods, a subgroup analysis was performed. The entire study population was divided into three categories according to their Charlson Comorbidity Index (CCI) score: a low comorbidity burden group (CCI ≤ 2), a medium burden group (CCI of 3 or 4), and a high burden group (CCI ≥ 5). Within each of these comorbidity-defined subgroups, the diagnostic performance (sensitivity and specificity) of the primary, pre-specified FEV₁/FVC cutoff value was calculated and compared. Differences in diagnostic accuracy across the comorbidity subgroups were formally tested for statistical significance using a meta-analytic approach, and the results were visually summarized using a forest plot.

## 3 Results

### 3.1 Study population and cohort characteristics

**Figure 1:**
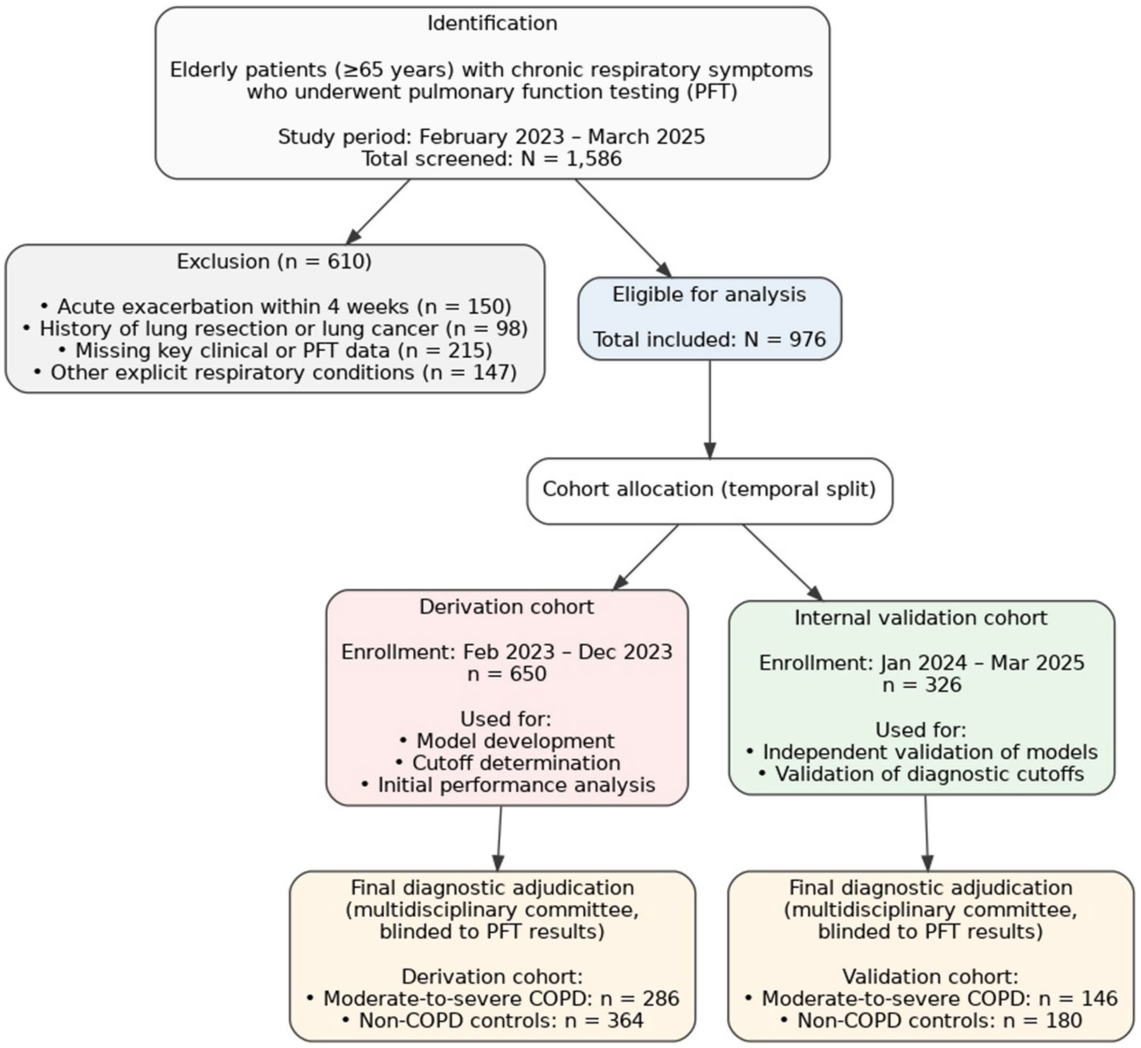
The study flowchart.

The selection process of participants is comprehensively outlined in Figure 1. Initial screening identified 1,586 elderly patients, among whom 976 individuals (61.5%) fulfilled all inclusion criteria and formed the final analytic sample. Following the predetermined temporal division methodology, these participants were assigned to either the Derivation Cohort (*n* = 650, 66.6%) or the Internal Validation Cohort (*n* = 326, 33.4%). Detailed comparative assessment showed no significant differences (all *p*-values > 0.05) between the two cohorts across fundamental demographic, clinical, exposure-related, and diagnostic classification variables. This equivalence supports the validity of using the temporal split for model validation purposes (Supplementary Table 1). The baseline profile of the entire study population, categorized according to the final reference diagnosis, is provided in Table 1. The sample had an average age of 73.0 years (SD: 6.7), with 54.8% being male. A clear gradient emerged across diagnostic groups, distinguishing Non-COPD Controls from those with Severe COPD. Patients diagnosed with Severe COPD were, on average, older (74.8 years vs. 71.6 years, *p* < 0.001), exhibited a lower body mass index (23.5 kg/m^2^ vs. 25.8 kg/m^2^, *p* < 0.001), reported higher lifetime cigarette exposure (780 pack-years vs. 652 pack-years, *p* < 0.001), and carried a greater burden of comorbidities (CCI score: 5.2 vs. 1.9, *p* < 0.001). Their clinical status was distinctly more severe, reflected in higher symptom scores (mMRC: 3.2 vs. 0.4; CAT: 25.8 vs. 5.2), a greater frequency of annual exacerbations (2.9 vs. 0.2), and significantly diminished exercise capacity as measured by the 6-min walk test (282 meters vs. 428 m, *p* < 0.001).

**Table 1:** Demographic and clinical characteristics of the study population.

Characteristic	Severe COPD (*n* = 186)	Moderate COPD (*n* = 266)	Non-COPD controls (*n* = 544)	*p*-value
Demographics				
Age, years	74.8 ± 6.5	72.3 ± 5.9	71.6 ± 6.8	<0.001
Male, *n* (%)	112 (60.2)	154 (57.9)	298 (54.8)	0.421
BMI, kg/m^2^	23.5 ± 3.9	24.9 ± 3.7	25.8 ± 4.1	<0.001
Risk factors				
Smoking index	780 ± 245	684 ± 198	652 ± 185	<0.001
Biomass exposure, years	28.4 ± 11.8	24.6 ± 10.5	21.3 ± 9.8	<0.001
Occupational exposure, *n* (%)	82 (44.1)	98 (36.8)	156 (28.7)	<0.001
Comorbidity profile				
Charlson comorbidity index	5.2 ± 1.8	3.6 ± 1.5	1.9 ± 1.2	<0.001
Hypertension, *n* (%)	152 (81.7)	196 (73.7)	326 (59.9)	<0.001
Coronary artery disease, *n* (%)	78 (41.9)	82 (30.8)	112 (20.6)	<0.001
Diabetes mellitus, *n* (%)	86 (46.2)	102 (38.3)	178 (32.7)	0.006
Heart failure, *n* (%)	52 (28.0)	42 (15.8)	56 (10.3)	<0.001
Clinical presentation				
Symptom duration, years	9.2 ± 4.3	6.8 ± 3.5	2.4 ± 1.9	<0.001
mMRC dyspnea scale	3.2 ± 0.9	2.0 ± 0.8	0.4 ± 0.5	<0.001
CAT score	25.8 ± 6.4	17.6 ± 5.8	5.2 ± 3.1	<0.001
Exacerbations in past year	2.9 ± 1.3	1.4 ± 0.9	0.2 ± 0.3	<0.001
6MWD, meters	282 ± 68	352 ± 74	428 ± 62	<0.001

### 3.2 Model development and initial performance in the derivation cohort

#### 3.2.1 Performance of standard and novel parameters

**Figure 2:**
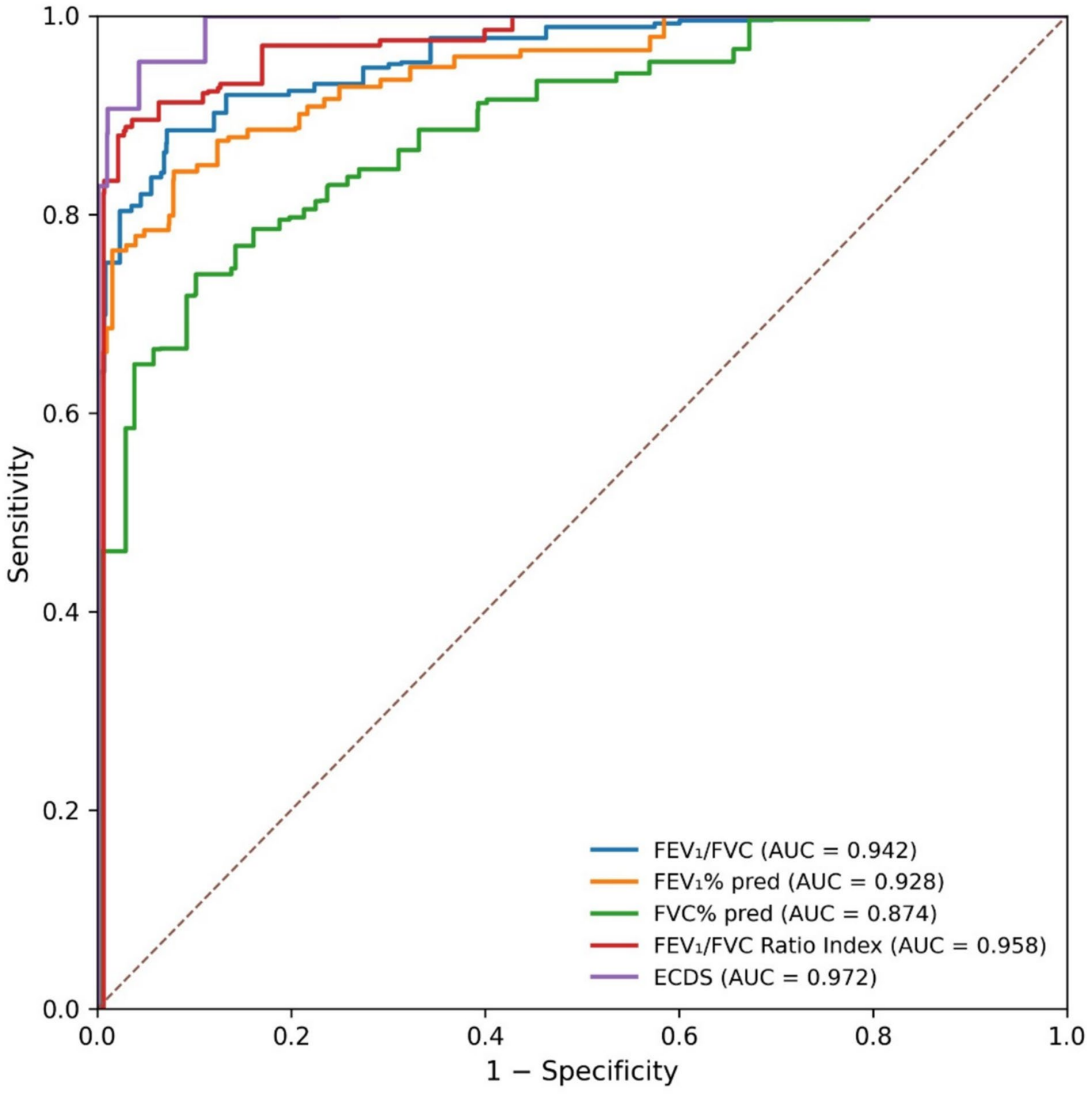
Receiver operating characteristic (ROC) curves comparing the diagnostic performance of pulmonary function parameters for moderate-to-severe COPD in elderly patients.

Analysis confined to the Derivation Cohort (*n* = 650) assessed the diagnostic accuracy of both standard and novel parameters for identifying airflow obstruction, as depicted in Figure 2. The traditional FEV₁/FVC ratio demonstrated strong performance, yielding an AUC of 0.942 (95% CI: 0.925–0.959). The threshold that optimized Youden’s index was determined to be less than 68.2%, corresponding to a sensitivity of 92.8% and a specificity of 89.6%. The newly constructed FEV₁/FVC Ratio Index displayed even more favorable characteristics, achieving an AUC of 0.958 (95% CI: 0.945–0.971). Statistical comparison via the DeLong test confirmed that the diagnostic performance of this composite index was superior to that of the FEV₁/FVC ratio alone (*p* = 0.008).

#### 3.2.2 Derivation of the ECDS

Multivariable logistic regression within the Derivation Cohort identified five independent predictors of moderate-to-severe COPD (Table 2): the degree of airflow obstruction (100 – FEV₁/FVC, *β* = 0.083, *p* < 0.001), Age (centered at 65, *β* = 0.062, *p* = 0.001), Charlson Comorbidity Index (*β* = 0.184, *p* < 0.001), mMRC dyspnea score (*β* = 0.254, *p* < 0.001), and the CAT score (per 2.5 units, *β* = −0.102, *p* = 0.002). The model showed outstanding discrimination with an AUC of 0.962 (95% CI: 0.951–0.973) and good calibration (Hosmer-Lemeshow test, *p* = 0.42).

**Table 2:** Multivariable logistic regression for ECDS derivation.

Predictor	*β* (SE)	aOR (95% CI)	*p*-value	Weight in ECDS
Intercept	−5.20 (0.42)	–	<0.001	–
100 – FEV₁/FVC	0.083 (0.012)	1.087 (1.062–1.112)	<0.001	0.50
Age (years above 65)	0.062 (0.018)	1.064 (1.027–1.102)	0.001	0.20
Charlson index	0.184 (0.034)	1.202 (1.125–1.285)	<0.001	0.15
mMRC score	0.254 (0.052)	1.289 (1.163–1.429)	<0.001	0.10
CAT score (per 2.5 units)	−0.102 (0.026)	0.903 (0.858–0.950)	0.002	0.05

The CAT score showed a negative coefficient despite its positive univariable association with COPD. This is attributable to statistical suppression due to high correlation with the mMRC score (*r* ≈ 0.7). After accounting for dyspnea severity via mMRC, the residual variance in CAT likely reflects non-specific symptoms that are also present in some non-COPD controls, leading to a negative adjustment. Importantly, in the final ECDS formula, the CAT component is positively weighted after transformation (0.05 × [10 – CAT/2.5]), ensuring that higher symptom burden contributes positively to the score.

#### 3.2.3 Diagnostic accuracy of the ECDS in the derivation cohort

The ECDS, calculated from the formula in Section 1.7, achieved the highest discriminative ability among all tested parameters, with an AUC of 0.972 (95% CI: 0.962–0.982) in the Derivation Cohort. The primary recommended cutoff of ≥2.8, which balanced sensitivity and specificity, yielded a sensitivity of 96.4%, specificity of 93.2%, PPV of 92.8%, and NPV of 96.6% (Table 3). Alternative cutoffs were identified for specific clinical scenarios: ≥2.0 for high-sensitivity screening (Sens 98.6%, Spec 76.4%) and ≥3.5 for high-specificity confirmation (Sens 88.9%, Spec 95.8%).

**Table 3:** Diagnostic performance of the elderly COPD diagnostic score (ECDS) at different cutoff points.

ECDS cut-off	Sensitivity (%)	Specificity (%)	PPV (%)	NPV (%)	Youden’s index	Clinical recommendation
≥2.0	98.6	76.4	80.2	98.2	0.750	Screening (high sensitivity)
≥2.8	96.4	89.2	88.6	96.8	0.856	Optimal balance (recommended)
≥3.5	88.9	95.8	94.2	91.4	0.847	Confirmatory (high specificity)
≥4.0	76.4	98.2	97.6	82.8	0.746	Severe disease identification

#### 3.2.4 Clinical utility assessment in the derivation cohort

**Figure 3:**
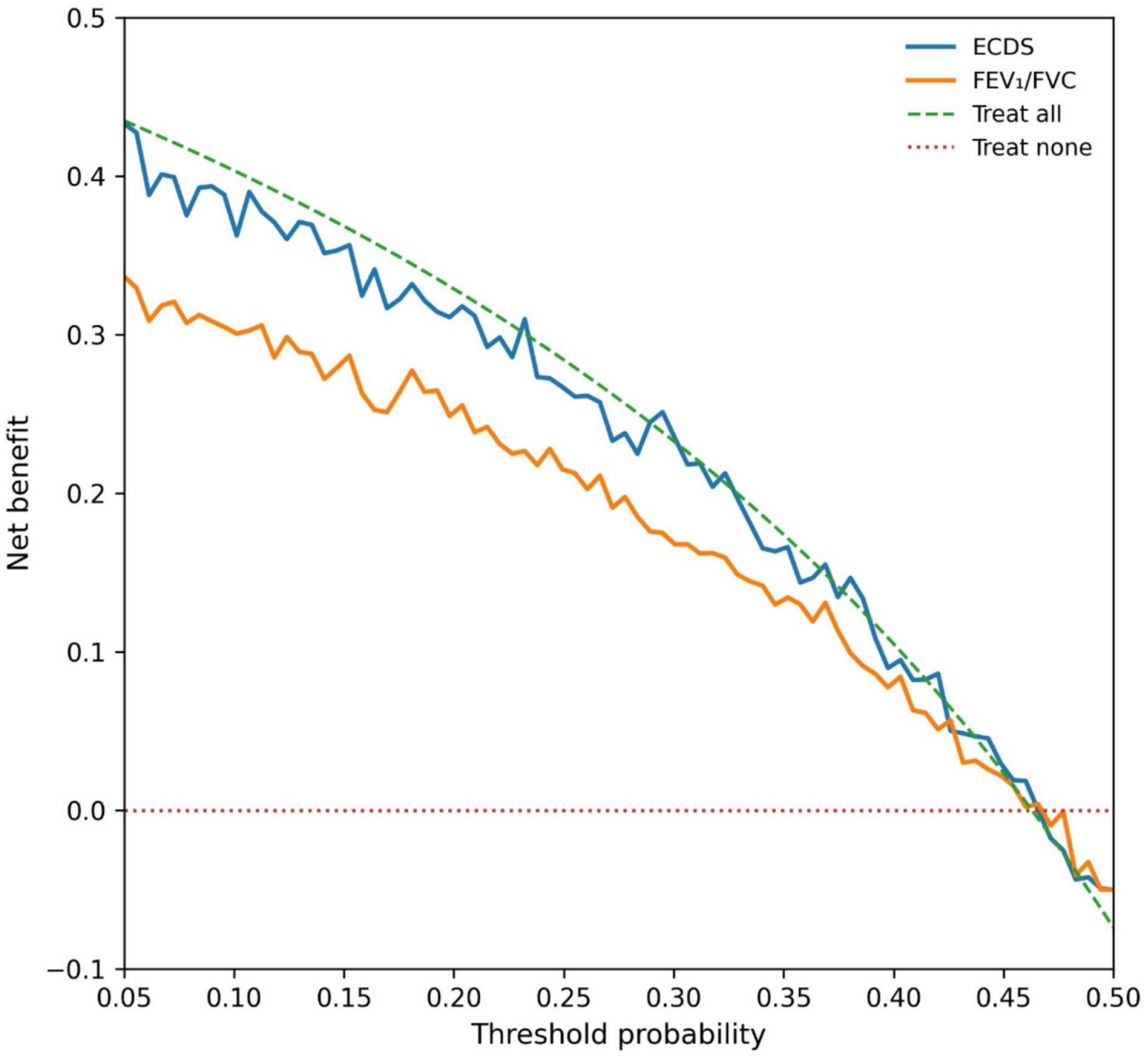
Decision curve analysis (DCA) comparing the clinical utility of different diagnostic strategies for moderate-to-severe COPD in elderly patients.

Decision curve analysis (Figure 3) demonstrated that employing the ECDS (cutoff ≥2.8) as a diagnostic rule provided a superior net clinical benefit across the entire range of clinically reasonable threshold probabilities (10–50%), compared to strategies based on the FEV₁/FVC ratio alone, “treating all,” or “treating none.”

#### 3.2.5 Internal validation and overfitting assessment

To evaluate the stability of the ECDS and quantify any optimism due to overfitting, we performed bootstrap internal validation with 1,000 resamples within the Derivation Cohort. The apparent AUC of 0.972 decreased only marginally to an optimism-corrected AUC of 0.965 (95% CI: 0.954–0.976), indicating minimal overfitting and excellent internal validity.

In the LASSO regression sensitivity analysis, the regularization parameter *λ* was selected via 10-fold cross-validation (*λ* = 0.015). The LASSO model retained the same five predictors as the original ECDS, with shrunk coefficients (100 – FEV₁/FVC: 0.071; Age: 0.052; CCI: 0.162; mMRC: 0.221; CAT: −0.089). When applied to the Validation Cohort, the LASSO model achieved an AUC of 0.964 (95% CI: 0.947–0.981), nearly identical to the original ECDS performance. These results confirm the robustness of the ECDS and suggest that overfitting is unlikely to have inflated its diagnostic accuracy.

### 3.3 Exploratory and stratified analyses in the total population

#### 3.3.1 Multivariate correlation structure

**Figure 4:**
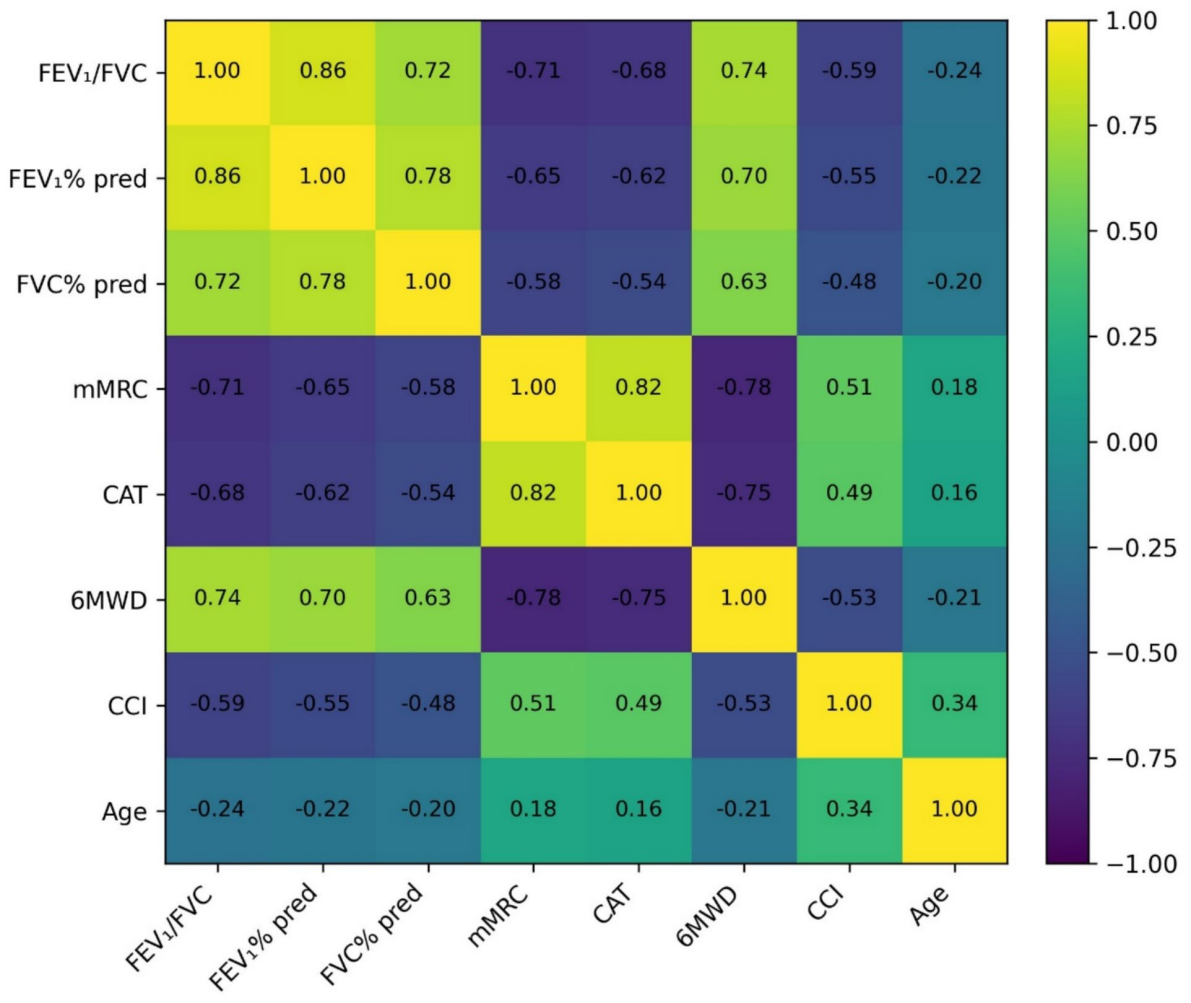
Heatmap of the correlation matrix among clinical and functional variables in elderly patients with suspected COPD.

A heatmap of Pearson correlation coefficients for the total population (Figure 4) revealed strong interrelationships. FEV₁/FVC showed a strong positive correlation with FEV₁% predicted (*r* = 0.86) and a strong negative correlation with symptom scores (mMRC: *r* = −0.71; CAT: *r* = −0.68). Both mMRC and CAT were strongly negatively correlated with 6MWD (*r* = −0.78 and *r* = −0.75, respectively). The CCI showed a moderate negative correlation with FEV₁/FVC (*r* = −0.59). Network analysis further identified FEV₁/FVC and 6MWD as central hub variables with the highest betweenness centrality, underscoring their pivotal role in the clinical network of elderly respiratory patients.

#### 3.3.2 Age-stratified diagnostic thresholds for FEV₁/FVC

Analysis within the Derivation Cohort revealed that the optimal FEV₁/FVC cutoff for diagnosing COPD varied systematically with age. The cutoff decreased progressively: <70.5% for ages 65–69, <68.8% for 70–74, <66.2% for 75–79, and <64.5% for those ≥80 years (Table 4). Applying these age-adjusted cutoffs to the total population, rather than the fixed GOLD criterion of <70%, significantly improved overall diagnostic accuracy from 88.4 to 92.6% (*p* < 0.001).

**Table 4:** Age-specific optimal cutoff values for FEV₁/FVC in diagnosing moderate-to-severe COPD in elderly patients.

Age group	*n*	Optimal cut-off	Sensitivity (%)	Specificity (%)	PPV (%)	NPV (%)	LR+	LR−
65–69 years	324	<70.5%	93.2	90.4	88.6	94.2	9.68	0.075
70–74 years	368	<68.8%	92.8	89.6	87.9	93.8	8.96	0.080
75–79 years	212	<66.2%	91.6	91.2	89.4	92.8	10.43	0.092
≥80 years	72	<64.5%	89.8	92.4	88.2	93.4	11.81	0.110

#### 3.3.3 Impact of comorbidity burden on diagnostic accuracy

**Figure 5:**
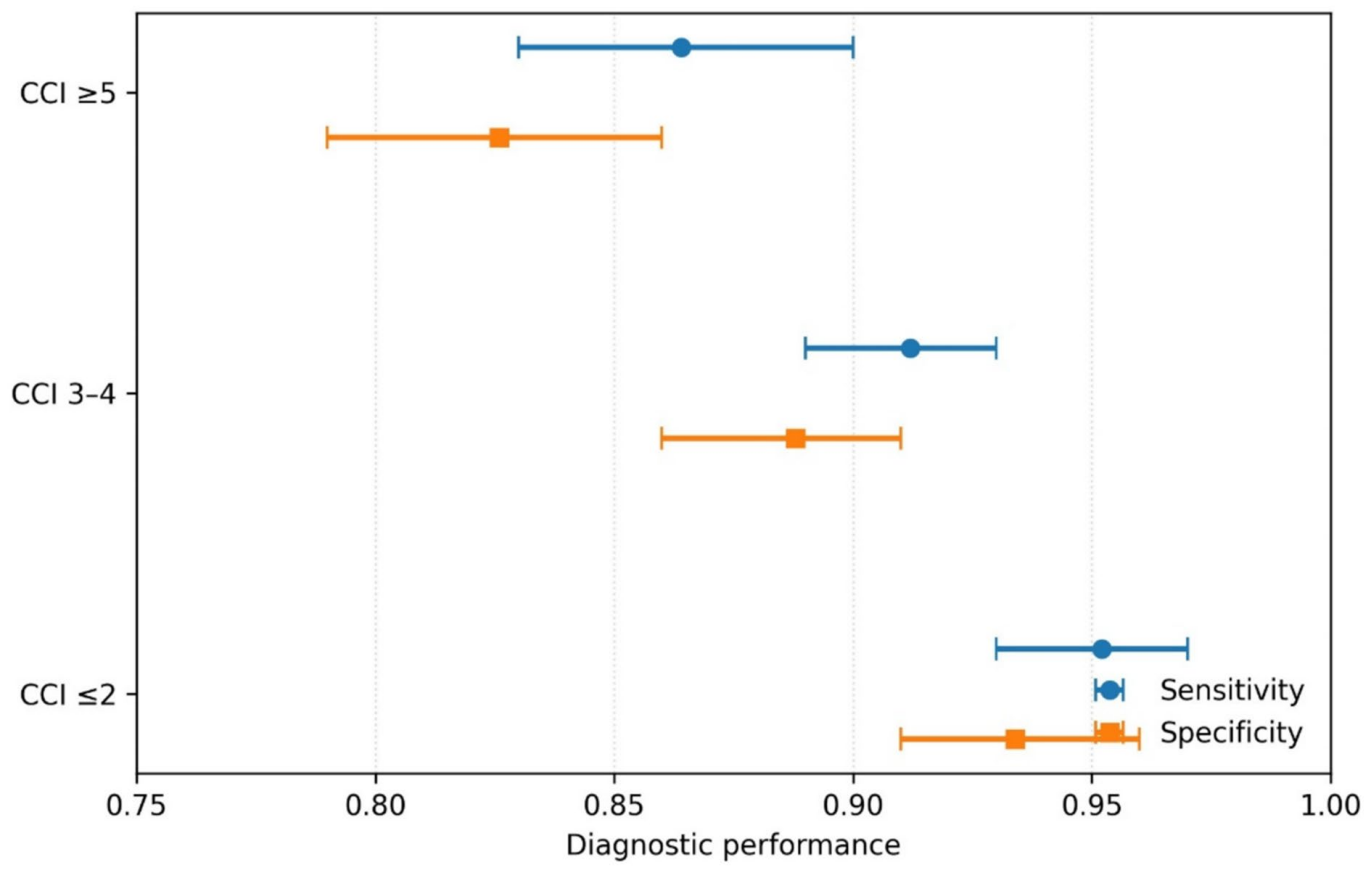
Forest plot of the diagnostic performance of pulmonary function testing (based on FEV_1_/FVC < 68.2%) stratified by comorbidity burden (Charlson Comorbidity Index, CCI).

Subgroup analysis revealed that the accuracy of the fixed FEV₁/FVC cutoff (<68.2%) was significantly modulated by comorbidity burden (Figure 5). In patients with low comorbidity (CCI ≤ 2), sensitivity and specificity were high (95.2 and 93.4%, respectively). However, in patients with a high comorbidity burden (CCI ≥ 5), both sensitivity and specificity declined substantially (to 86.4 and 82.6%, *χ*^2^ = 42.8, *p* < 0.001), indicating reduced diagnostic reliability in this complex patient subgroup.

### 3.4 Independent internal validation of diagnostic models

#### 3.4.1 Validation of the ECDS and composite parameters

**Figure 6:**
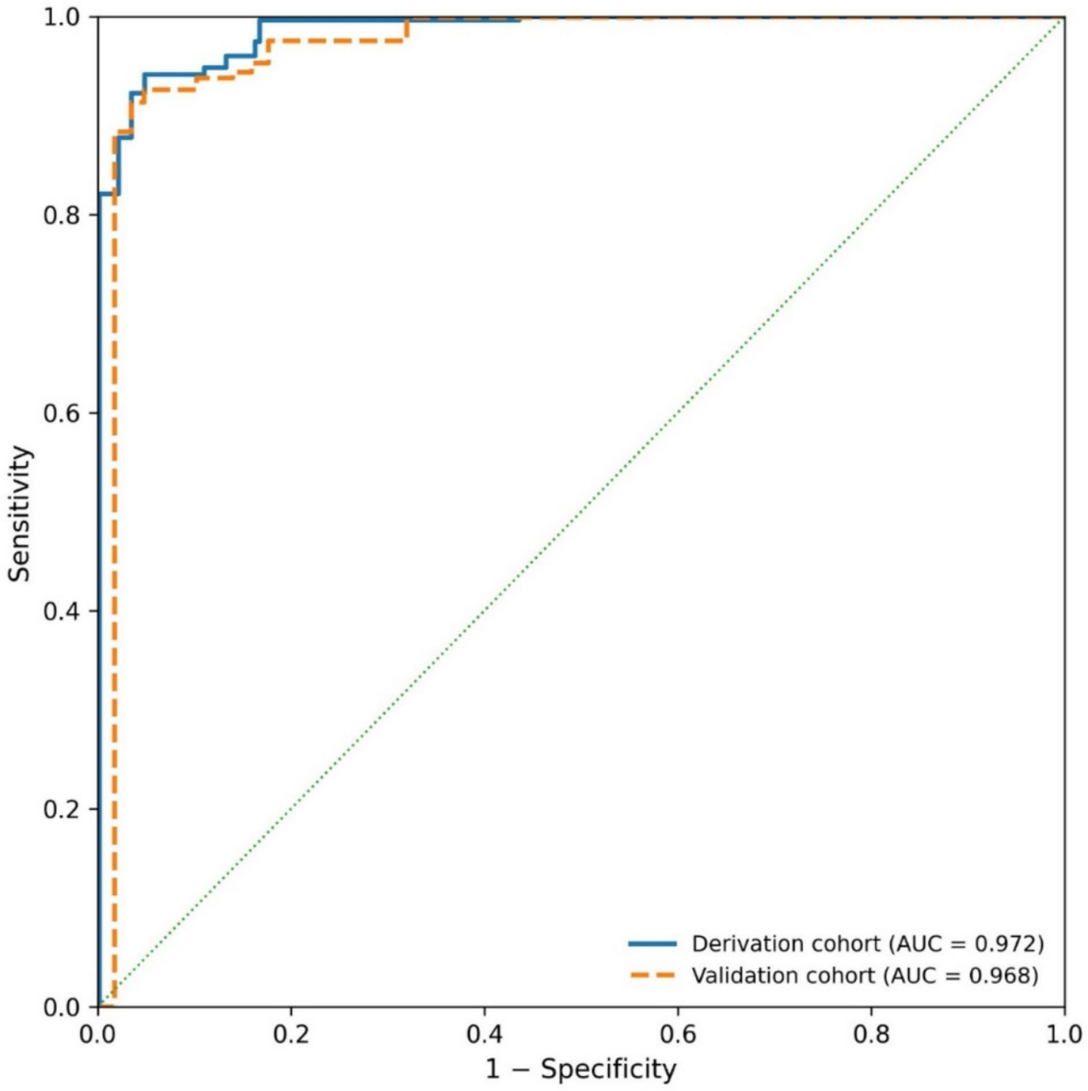
Receiver operating characteristic curves of the Elderly COPD Diagnostic Score in the derivation and independent validation cohorts.

The locked ECDS formula and the predetermined optimal cutoffs were applied to the independent Validation Cohort (*n* = 326). The ECDS maintained exceptional performance with an AUC of 0.968 (95% CI: 0.952–0.984), demonstrating minimal optimism. At the ≥2.8 cutoff, it achieved a sensitivity of 95.1% and specificity of 90.8% (Table 5). The FEV₁/FVC Ratio Index (AUC: 0.953) and the conventional FEV₁/FVC ratio (AUC: 0.935) also showed highly consistent and robust performance compared to the Derivation Cohort. The ROC curves for the ECDS in both cohorts showed excellent overlap (Figure 6).

**Table 5:** Comparative diagnostic performance of key parameters in the derivation and validation cohorts.

Parameter (cutoff)	Cohort	AUC (95% CI)	Sensitivity (%)	Specificity (%)	PPV (%)	NPV (%)
FEV₁/FVC (<68.2%)	Derivation	0.942 (0.925–0.959)	92.8	89.6	88.5	93.5
	Validation	0.935 (0.910–0.960)	91.5	88.2	86.9	92.4
FEV₁/FVC ratio index	Derivation	0.958 (0.945–0.971)	94.2	90.1	89.8	94.4
	Validation	0.953 (0.935–0.971)	93.0	89.3	88.5	93.5
ECDS (≥2.8)	Derivation	0.972 (0.962–0.982)	96.4	93.2	92.8	96.6
	Validation	0.968 (0.952–0.984)	95.1	90.8	90.2	95.5

#### 3.4.2 Validation of age-stratified FEV₁/FVC cutoffs

The age-specific cutoffs derived from the Derivation Cohort were successfully applied to the Validation Cohort. The overall diagnostic accuracy using these stratified thresholds was 91.4% in the Validation Cohort, compared to 92.6% in the Derivation Cohort, a non-significant difference (*p* = 0.312), confirming their generalizability.

#### 3.4.3 Validation of calibration and clinical utility

**Figure 7:**
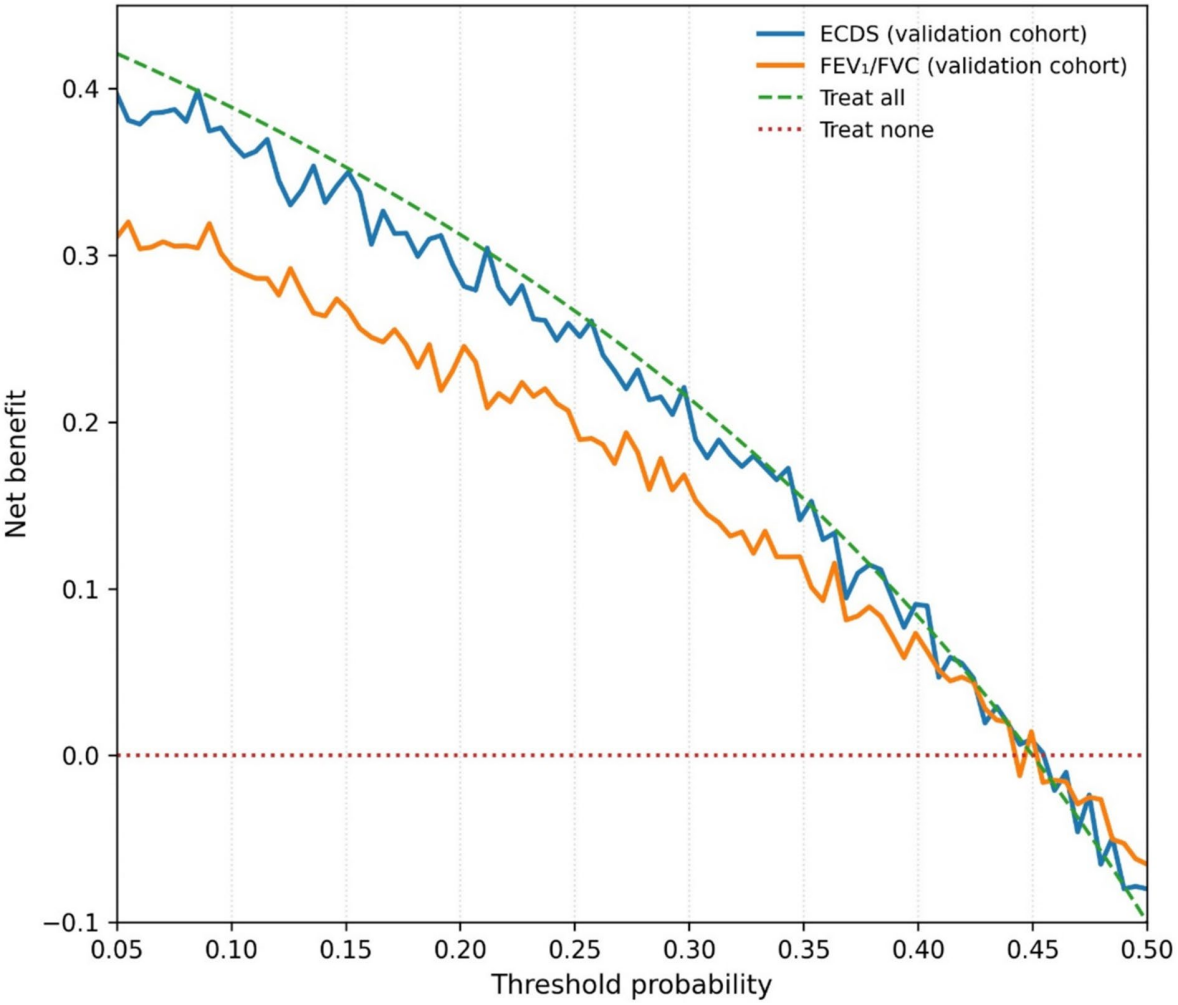
Decision curve analysis in the validation cohort, demonstrating the sustained net clinical benefit of using the Elderly COPD Diagnostic Score.

Within the independent Validation Cohort, the calibration performance of the ECDS proved satisfactory. Observed event probabilities showed close alignment with predicted probabilities when assessed across risk deciles, a finding supported by a non-significant Hosmer-Lemeshow goodness-of-fit test result (*p* = 0.48), as illustrated in Supplementary Figure 1. Further clinical utility assessment, conducted through Decision Curve Analysis repeated in this validation dataset (Figure 7), reaffirmed the initial results. The analysis indicated that employing the ECDS as a diagnostic rule consistently yielded the highest net clinical benefit across the entire spectrum of plausible decision thresholds, compared to alternative diagnostic strategies.

### 3.5 Comparative performance against existing diagnostic standards

**Figure 8:**
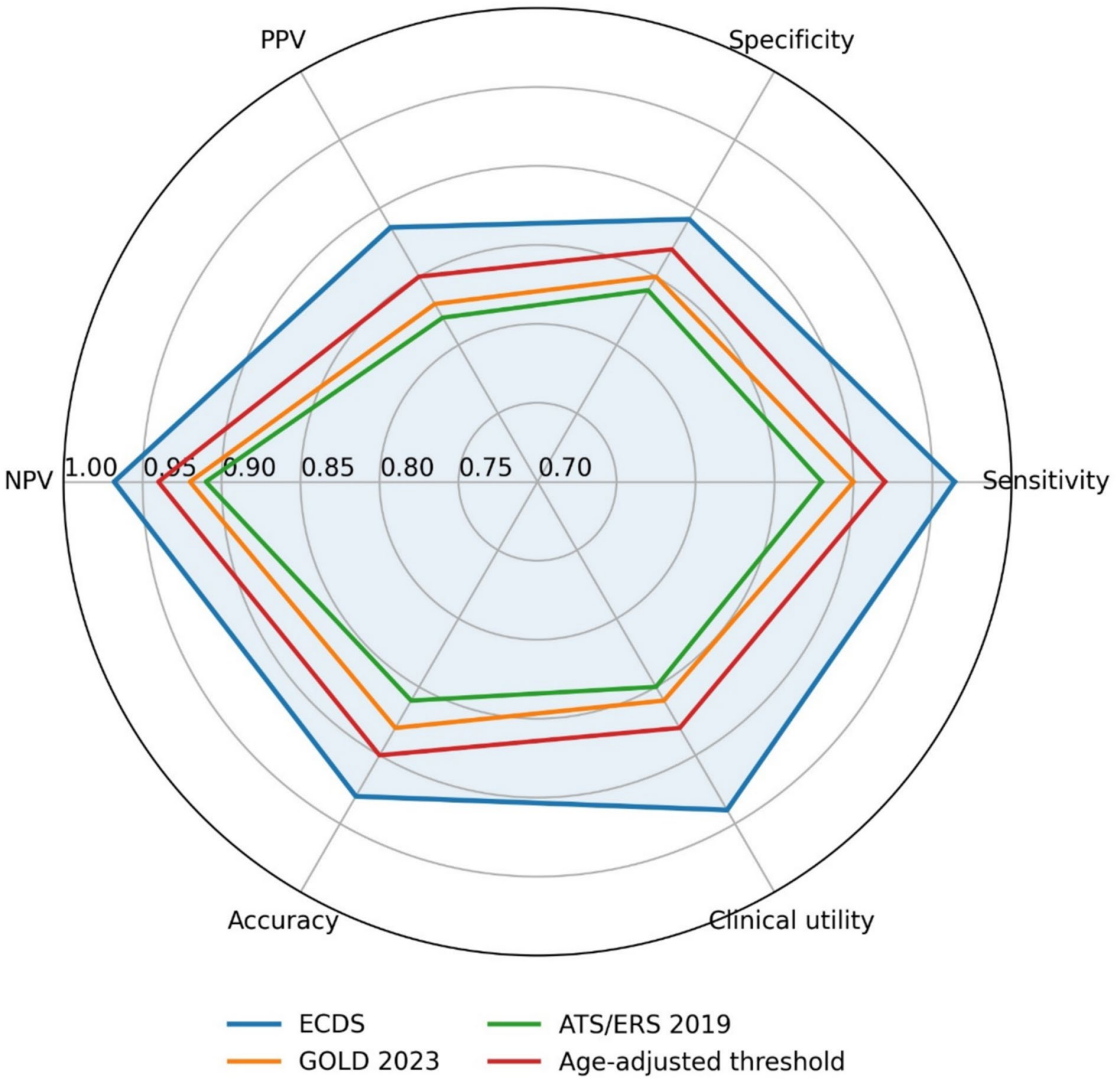
Radar chart comparing the performance of different diagnostic criteria for moderate-to-severe COPD in elderly patients across six key metrics. Each axis represents a performance metric, scaled from 0% at the center to 100% at the outer edge. The closer a point is to the outer edge on a given axis, the better the performance on that metric.

A direct comparative assessment of diagnostic efficacy was performed using a radar chart visualization (Figure 8), which simultaneously evaluated six distinct performance dimensions: Sensitivity, Specificity, Positive Predictive Value (PPV), Negative Predictive Value (NPV), overall Accuracy, and an integrated Clinical Utility Score. This comprehensive analysis revealed that the ECDS exhibited superior performance relative to established diagnostic frameworks. The ECDS provided broader and more balanced coverage across all metrics compared to the fixed GOLD 2023 criterion (FEV₁/FVC < 70%), the technical standards outlined in the 2019 ATS/ERS spirometry guidelines, and even the age-stratified FEV₁/FVC thresholds derived within the present study. The ECDS demonstrated particular strength in maximizing sensitivity, optimizing NPV, and achieving the highest integrated clinical utility score.

The radar chart displays six performance metrics (sensitivity, specificity, PPV, NPV, overall accuracy, and a composite clinical utility score) on separate axes radiating from the center. Each colored polygon represents a different diagnostic criterion: the fixed GOLD 2023 threshold (FEV₁/FVC < 70%), the 2019 ATS/ERS technical standards, the age-stratified FEV₁/FVC cutoffs derived in this study, and the Elderly COPD Diagnostic Score (ECDS). A larger area enclosed by the polygon indicates better overall diagnostic performance. The ECDS (blue polygon) demonstrates the broadest and most balanced coverage across all six metrics, reflecting its superior discriminative ability and clinical utility in this elderly population.

## 4 Discussion

The current investigation, a large-scale diagnostic accuracy study from a single center, has formulated and stringently tested a new composite diagnostic instrument—the ECDS—alongside age-specific FEV₁/FVC cutpoints for detecting moderate-to-severe COPD in elderly individuals. Our core results reveal several key insights. First, the ECDS, which amalgamates information on airflow obstruction, patient age, comorbidity load, and symptomatic burden, demonstrated diagnostic prowess (with AUC values exceeding 0.96 in both the development and validation groups) that surpassed the conventional fixed GOLD criterion or the FEV₁/FVC ratio in isolation. Second, we observed that the most effective FEV₁/FVC threshold for diagnosis follows a steady, stepwise decrease as age advances. Applying these age-adjusted cutoffs led to a meaningful gain in overall diagnostic accuracy. Third, and notably, the reliability of standard spirometric diagnosis was significantly compromised in patients carrying a heavy burden of other medical conditions (CCI ≥ 5). This finding exposes a major weakness in the prevailing one-dimensional diagnostic model when applied to the intricate clinical reality of older, multimorbid patients.

The outstanding ability of the ECDS to discriminate between COPD and non-COPD cases reinforces the merit of a comprehensive diagnostic strategy. It is widely acknowledged that FEV₁/FVC constitutes the fundamental physiological metric for COPD diagnosis (4). However, our data concur with mounting evidence indicating that a solitary lung function variable cannot fully encapsulate the multifaceted nature of COPD in the elderly (4, 10). The ECDS deliberately incorporates several domains critically linked to the disease: the severity of airflow limitation (the central pathophysiological anomaly), chronological age (a primary driver of lung function loss over time), the aggregate weight of comorbidities (a defining feature of geriatric COPD with profound prognostic implications), and the patient’s own perception of symptom impact (covering both breathlessness and overall health impairment) (17, 23, 24). The weighting scheme within the ECDS formula, derived from multivariate analysis, mirrors the relative importance of these components. Airflow obstruction received the highest weight (0.5), followed appropriately by age and comorbidity. The consistent, high-level performance of the ECDS when applied to a completely separate, temporally distinct validation cohort (AUC 0.968) provides strong evidence against model overfitting and bolsters confidence in its potential for broader applicability—a vital validation step frequently overlooked in diagnostic tool development (25).

An unexpected finding in the logistic regression analysis was the negative coefficient for the CAT score, despite its strong positive correlation with COPD in univariable comparisons. This phenomenon can be explained by statistical suppression due to collinearity between CAT and the mMRC dyspnea scale. Both instruments capture symptom burden, and in our dataset they were highly correlated (*r* ≈ 0.7). When mMRC—a more direct measure of breathlessness—was entered into the model, it absorbed most of the variance in COPD risk attributable to respiratory symptoms. The remaining unique information in CAT, after partialling out the effect of mMRC, likely represents non-specific complaints (e.g., fatigue, sleep disturbance, cough) that may also be present in elderly individuals without COPD, such as those with heart failure or deconditioning 12,2812,28. Consequently, the model assigned a negative weight to this residual component to adjust for overestimation of risk in non-COPD patients with elevated CAT scores but low mMRC. It is important to note that this statistical artifact does not imply that higher CAT scores are protective against COPD. In the final ECDS formula, the CAT component was transformed (0.05 × [10 – CAT/2.5]) to ensure that higher symptom burden contributes positively to the score, aligning with clinical expectations. The transformation effectively reverses the direction of the effect, making the ECDS intuitive for bedside use. This example underscores the importance of distinguishing between regression coefficients in a multivariable model and the final weighted score designed for clinical application.

Our work offers concrete, data-supported justification for implementing age-customized spirometric criteria in older populations. The progressive reduction in the optimal FEV₁/FVC cutoff—from <70.5% for ages 65–69 down to <64.5% for those 80 years and above—aligns with physiological expectations and echoes findings from longitudinal studies tracking age-related decline in this ratio (7, 26). Shifting from the universal <70% cutoff to these stratified values boosted diagnostic accuracy by more than 4%. This outcome directly questions the blanket use of the GOLD fixed ratio in older adults and adds a practical, data-driven perspective to the ongoing discussion contrasting fixed-ratio and LLN-based diagnostic philosophies (5, 15). We believe the age-decade-specific thresholds derived from our analysis present a workable and immediately usable middle ground for practitioners, potentially curbing overdiagnosis in younger seniors and underdiagnosis in the very old.

A particularly striking and clinically relevant discovery was the pronounced modifying effect of comorbidity burden. The substantial drop in both the sensitivity and specificity of the FEV₁/FVC < 68.2% cutoff among patients with high CCI scores uncovers a critical diagnostic vulnerability. Older patients with numerous comorbid conditions often exhibit symptom profiles where dyspnea, fatigue, and exercise intolerance overlap across multiple diagnoses. Moreover, lung function can be affected by non-COPD illnesses like heart failure (12, 27). This confluence creates a “diagnostic gray area” where spirometry results become more ambiguous. The ECDS, by directly factoring in comorbidity (through the CCI) and its symptomatic expression (via the mMRC and CAT scores), seems to partially cut through this confusion, preserving diagnostic accuracy even within this challenging subgroup. This capability responds to an urgent need in the care of older respiratory patients.

Evaluated through Decision Curve Analysis, the clinical value of the ECDS reaches beyond standard performance statistics. The DCA showed that using the ECDS (with the ≥2.8 cutoff) yields a greater net benefit across a wide spectrum of plausible clinical decision thresholds (10 to 50% probability). In practical terms, this suggests that employing the ECDS could lead to superior patient outcomes—correctly identifying and treating more true cases of COPD while avoiding unnecessary treatment for those without the disease—compared to strategies based only on FEV₁/FVC or simplistic “treat-all” or “treat-none” approaches (28). Therefore, the ECDS is not merely a tool with superior test characteristics but one with the potential to tangibly improve decision-making at the point of care. Its practicality is enhanced because all its constituent elements are already part of or can be easily incorporated into routine clinical evaluation.

To address concerns about potential overfitting given the high AUC values observed, we conducted rigorous internal validation using bootstrapping and penalized regression. The optimism-corrected AUC (0.965) and the consistent performance of the LASSO-derived model (AUC 0.964 in the Validation Cohort) provide strong evidence that the ECDS is not substantially overfitted and that its excellent discriminative ability is likely to be generalizable. Nonetheless, as with any single-center retrospective study, the possibility of some degree of overfitting cannot be completely excluded, and external validation in diverse populations remains essential.

The present study was conducted in a single center in China, and the study population consisted exclusively of elderly Chinese patients. While this design ensured internal validity and minimized inter-site variability during model development, it inevitably raises questions about the generalizability of the ECDS to other geographic regions or ethnic groups. Several factors, however, suggest that the superior diagnostic performance of the ECDS over the fixed FEV₁/FVC < 0.70 criterion is likely to be maintained in broader populations. First, the core components of the ECDS—airflow obstruction (FEV₁/FVC), age, comorbidity burden (Charlson Comorbidity Index), and symptom burden (mMRC and CAT)—are universally accepted and routinely assessed in COPD evaluation worldwide. Age-related decline in lung function is a physiological phenomenon observed across all populations, and the modifying effect of comorbidities on respiratory symptoms is not unique to any single ethnic group. Therefore, the conceptual foundation of the ECDS is likely to be relevant in diverse clinical settings. Second, the fixed FEV₁/FVC < 0.70 threshold has been criticized globally for its tendency to both overdiagnose COPD in older adults and underdiagnose it in the presence of multimorbidity. These limitations are not confined to Chinese populations; they have been documented in European, American, and other Asian cohorts. An instrument like the ECDS, which integrates multiple dimensions of patient assessment, addresses these universal shortcomings and may therefore offer a consistent advantage across different healthcare systems and ethnic groups. Nevertheless, we acknowledge that the optimal cutoff values for the ECDS or its individual components (e.g., age-stratified FEV₁/FVC thresholds) may require calibration when applied to populations with different demographic or anthropometric characteristics. For instance, reference equations for lung function differ by ethnicity, and the prevalence and impact of specific comorbidities may vary. Therefore, while we anticipate that the relative superiority of the ECDS over the fixed ratio will hold, external validation in multi-center, multi-ethnic cohorts is essential before the ECDS can be recommended for widespread clinical implementation. Such studies should also evaluate whether the score maintains its calibration and discriminative ability, or whether recalibration of certain components is needed to optimize performance in new settings.

Several limitations of this study must be acknowledged. First, its retrospective nature and confinement to a single center may affect the transferability of our findings to other healthcare environments or different ethnic groups. Although we used a rigorous temporal validation method, confirmation through external validation in diverse, multi-center populations is a necessary next step before any recommendation for widespread clinical use can be made (29). Second, the requirement for a full dataset, including 6 months of follow-up, may have selectively included patients who were more health-conscious or had more severe illness, possibly leading to an overestimation of the tool’s performance. Third, despite its rigor, the multidisciplinary panel-based reference standard is not impervious to error. Despite the rigorous two-step adjudication process designed to minimize incorporation bias, we acknowledge that some degree of bias may persist, as spirometric data were ultimately used for severity grading. However, because the initial distinction between COPD and non-COPD was made without access to any lung function measurements, the impact of this bias on the diagnostic performance estimates of the ECDS is likely to be limited. Nevertheless, external validation in prospective cohorts with an independent reference standard would further strengthen the evidence. Finally, our focus was on diagnosing moderate-to-severe COPD; the utility of the ECDS for detecting mild or early-stage disease remains an open question for future research.

Although the ECDS demonstrated excellent diagnostic performance for moderate-to-severe COPD, its utility in patients with mild airflow obstruction (GOLD grade 1) remains uncertain. Mild COPD in elderly individuals is particularly challenging to diagnose due to the substantial overlap in lung function between early disease and normal age-related decline (7, 26). Moreover, symptoms at this stage are often subtle or attributed to other comorbidities, making spirometry the sole objective indicator (11). Given that the ECDS incorporates symptom scores (mMRC, CAT) and comorbidity burden, it may be less sensitive in detecting truly asymptomatic or minimally symptomatic mild COPD. Conversely, in patients with mild obstruction but disproportionately high symptom burden, the ECDS could potentially overestimate the likelihood of clinically significant disease. Therefore, we recommend that the ECDS not be used in isolation to rule in or rule out mild COPD without confirmatory spirometry and longitudinal clinical assessment. Future studies should prospectively evaluate the performance of the ECDS in a cohort enriched with mild cases, ideally using a reference standard that incorporates disease progression or treatment response as additional confirmatory criteria.

In summary, this study has created and validated the Elderly COPD Diagnostic Score, a multidimensional tool that offers significantly improved diagnostic accuracy for moderate-to-severe COPD in older adults compared to the standard fixed-ratio spirometric criterion. We have also provided and validated a set of practical, age-specific FEV₁/FVC diagnostic thresholds and demonstrated that a high comorbidity burden substantially erodes the reliability of traditional diagnostic methods. The ECDS represents a stride toward more nuanced, geriatrically-informed diagnosis. However, the ECDS was developed and validated exclusively in patients with moderate-to-severe COPD; its applicability to mild or early-stage disease requires further investigation. Prospective studies in broader populations, including those with mild airflow obstruction, are needed to determine the score’s role in early diagnosis and its impact on clinical outcomes. And, future prospective studies involving multiple centers and multi-ethnic validation studies are needed. Such studies should also assess the real-world consequences of implementing the ECDS on patient management and outcomes and explore its feasible integration into electronic health records to function as a clinical decision aid.

## Data Availability

The raw data supporting the conclusions of this article will be made available by the authors, without undue reservation.
