# Supplementary material for: Development and internal validation of the elderly COPD diagnostic score (ECDS): a multidimensional diagnostic tool for moderate-to-severe chronic obstructive pulmonary disease
Source: Front Med (Lausanne). 2026 Mar 27;13:1770654. doi: 10.3389/fmed.2026.1770654 (PMC13067287; doi:10.3389/fmed.2026.1770654)
Supplement: Supplementary file 1 [file Data_Sheet_1.docx]

**Supplementary Table 1. Baseline Characteristics of the Derivation and Validation Cohorts**

| **Characteristic** | **Derivation Cohort (n=650)** | **Validation Cohort (n=326)** | **P-value** |
| --- | --- | --- | --- |
| **Demographics** |  |  |  |
| Age, years | 73.1 ± 6.7 | 72.8 ± 6.5 | 0.452 |
| Male, n (%) | 362 (55.7) | 185 (56.7) | 0.754 |
| BMI, kg/m² | 25.2 ± 4.1 | 24.9 ± 3.9 | 0.289 |
| **Risk Factors** |  |  |  |
| Smoking index | 698 ± 215 | 684 ± 208 | 0.361 |
| Biomass exposure, years | 23.8 ± 11.2 | 24.1 ± 10.8 | 0.703 |
| Occupational exposure, n (%) | 218 (33.5) | 105 (32.2) | 0.678 |
| **Comorbidity Profile** |  |  |  |
| Charlson Comorbidity Index | 3.2 ± 1.9 | 3.4 ± 2.0 | 0.128 |
| Hypertension, n (%) | 430 (66.2) | 221 (67.8) | 0.616 |
| Coronary artery disease, n (%) | 176 (27.1) | 92 (28.2) | 0.720 |
| Diabetes mellitus, n (%) | 240 (36.9) | 126 (38.7) | 0.592 |
| Heart failure, n (%) | 98 (15.1) | 52 (16.0) | 0.732 |
| **Clinical Presentation** |  |  |  |
| Symptom duration, years | 5.2 ± 4.0 | 5.4 ± 4.1 | 0.489 |
| mMRC dyspnea scale | 1.6 ± 1.2 | 1.7 ± 1.3 | 0.257 |
| CAT score | 13.8 ± 9.6 | 14.1 ± 9.8 | 0.661 |
| Exacerbations in past year | 1.2 ± 1.3 | 1.3 ± 1.4 | 0.297 |
| 6MWD, meters | 378 ± 86 | 374 ± 88 | 0.511 |
| **Final Diagnosis Group** |  |  |  |
| Non-COPD Controls, n (%) | 364 (56.0) | 180 (55.2) | 0.814 |
| Moderate COPD, n (%) | 178 (27.4) | 88 (27.0) | 0.895 |
| Severe COPD, n (%) | 108 (16.6) | 58 (17.8) | 0.648 |
| **Moderate-to-Severe COPD Combined, n (%)** | **286 (44.0)** | **146 (44.8)** |  |


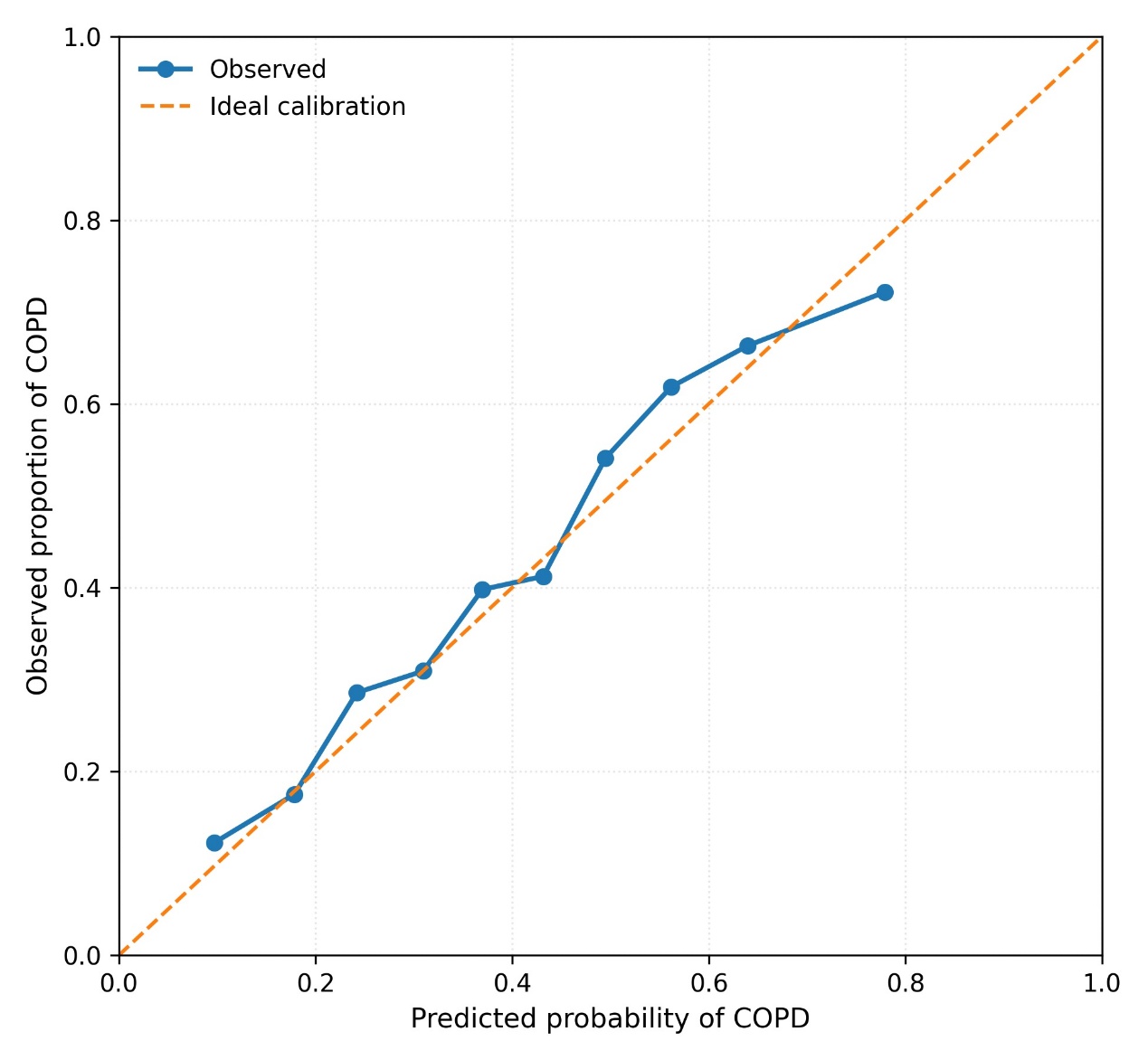


**Supplementary Figure 1. Calibration plot of the Elderly COPD Diagnostic Score (ECDS) in the validation cohort.**

The observed proportion of COPD across deciles of predicted risk closely aligned with the ideal calibration line, indicating good agreement between predicted and observed probabilities (Hosmer–Lemeshow test, P = 0.48).
